# Klotho expression in peripheral blood circulating cells is associated with vascular and systemic inflammation in atherosclerotic vascular disease

**DOI:** 10.1038/s41598-022-12548-z

**Published:** 2022-05-19

**Authors:** Ernesto Martín-Núñez, Atteneri Pérez-Castro, Víctor G. Tagua, Carolina Hernández-Carballo, Carla Ferri, Nayra Pérez-Delgado, Sergio Rodríguez-Ramos, Purificación Cerro-López, Ángel López-Castillo, Alejandro Delgado-Molinos, Victoria Castro López-Tarruella, Miguel A. Arévalo-Gómez, Ainhoa González-Luis, Alberto Martín-Olivera, Carmen Chaxiraxi Morales-Estévez, Carmen Mora-Fernández, Javier Donate-Correa, Juan F. Navarro-González

**Affiliations:** 1 Unidad de Investigación, Hospital Universitario Nuestra Señora de Candelaria, 38010 Santa Cruz de Tenerife, Tenerife Spain; 2 Escuela de Doctorado Y Estudios de Posgrado, Universidad de La Laguna, 38200 San Cristóbal de La Laguna, Tenerife Spain; 3 Instituto Universitario de Enfermedades Tropicales Y Salud Pública de Canarias, Universidad de La Laguna, 38200 San Cristóbal de La Laguna, Tenerife Spain; 4 Servicio de Análisis Clínicos, Hospital Universitario Nuestra Señora de Candelaria, 38010 Santa Cruz de Tenerife, Tenerife Spain; 5 Coordinación de Trasplantes, Hospital Universitario Nuestra Señora de Candelaria, 38010 Santa Cruz de Tenerife, Tenerife Spain; 6 Servicio de Cirugía Vascular, Hospital Universitario Nuestra Señora de Candelaria, 38010 Santa Cruz de Tenerife, Tenerife Spain; 7 Servicio de Anatomía Patológica, Hospital Universitario Nuestra Señora de Candelaria, 38010 Santa Cruz de Tenerife, Tenerife Spain; 8 Departamento de Anatomía E Histología Humana, Universidad de Salamanca, 37008 Salamanca, Spain; 9 Servicio de Nefrología, Hospital Universitario Nuestra Señora de Candelaria, 38010 Santa Cruz de Tenerife, Tenerife Spain; 10 Instituto de Tecnologías Biomédicas, Universidad de La Laguna, 38200 San Cristóbal de La Laguna, Tenerife Spain

**Keywords:** Atherosclerosis, Ageing

## Abstract

Cardiovascular disease is the leading cause of death worldwide. New therapeutic strategies are aimed to modulate the athero-inflammatory process that partially orchestrates underlying vascular damage. Peripheral blood circulating cells include different immune cells with a central role in the development of the atherogenic inflammatory response. The anti-aging protein α-Klotho has been related to protective effects against CVD. KL is expressed in monocytes, macrophages, and lymphocytes where it exerts anti-inflammatory effects. In this work, we analyse the relationships of the levels of inflammatory markers with the expression of the *KL* gene in PBCCs and with the serum levels of soluble KL in atherosclerotic vascular disease. For this, we conducted a cross-sectional single-center case–control study including a study group of 76 CVD patients and a control group of 16 cadaveric organ donors without medical antecedent or study indicating CVD. Vascular artery fragments and whole blood and serum samples were obtained during elective or organ retrieval surgery. Serum levels of sKL, TNFα and IL10, and gene expression levels of *KL, TNF, IL10, NFKB1*, *DNMT1,* and *DNMT3A* in PBCCs were measured. In these cells, we also determined *KL* promoter methylation percentage. Histological and immunohistochemical analyses were employed to visualize atherosclerotic lesions and to measure IL10 and TNFα levels in vascular fragments. Patients with CVD presented higher values of proinflammatory markers both at systemic and in the vasculature and in the PBCCs, compared to the control group. In PBCCs, CVD patients also presented lower gene expression levels of *KL* gene (56.4% difference, *P* < 0.001), higher gene expression levels of *DNMT1* and *DNMT3A* (*P *< 0.0001, for both) and a higher methylation status of in the promoter region of *KL* (34.1 ± 4.1% vs. 14.6 ± 3.4%, *P* < 0.01). In PBCCs and vasculature, *KL* gene expression correlated inversely with pro-inflammatory markers and directly with anti-inflammatory markers. sKL serum levels presented similar associations with the expression levels of pro- and anti-inflammatory markers in PBCCs. The differences in *KL* expression levels in PBCCs and in serum sKL levels with respect to control group was even greater in those CVD patients with macroscopically observable atheromatous plaques. We conclude that promoter methylation-mediated downregulation of *KL* gene expression in PBCCs is associated with the pro-inflammatory status in atherosclerotic vascular disease.

## Introduction

Atherosclerosis underlies most of cardiovascular diseases (CVD), such as coronary artery disease (CAD), stroke and peripheral artery disease (PAD)^1^. The pathophysiology of CVD constitutes a slow, progressive and chronic inflammatory process^2^ that results of the systemic influence of diverse cardiovascular risk factors (CVRF) -dyslipidemia, smoking, obesity, diabetes, and hypertension, among others-. These CVRF cause endothelial dysfunction and, thereby, permeabilization of low-density lipoproteins (LDL) and various inflammatory cells into subintimal space, ultimately leading to the atheroma plaque formation. Although conventional therapeutic strategies have been focused on managing these CVRF, new alternatives have been proposed focused on modulating the athero-inflammatory process^3,4^. Macrophages, monocytes, lymphocytes, and other peripheral blood circulating cells (PBCCs) have a central role in the development of the inflammatory response associated to the atherogenic process. Their contribution to this state range from the low-grade systemic inflammation that accompanies CVD (secretion of pro- or anti-inflammatory factors into the systemic circulation) to the resolution of the local response of the vascular wall (environmental signal transduction, uptake of LDL or LDLox particles, engulfment of dead cells, secretion of inflammatory cytokines or pro-resolving molecules, etc.)^2,5–7^. Immunomodulation of these cells constitutes an interesting approach to the development of new therapeutic strategies in CVD.

Deficits in the antiaging factor α-Klotho (KL) are observed in diverse pathologies related to human aging^8–13^. Reductions in the soluble form (sKL) have been associated with the appearance of CAD, PAD, heart failure or stroke, as well as with poor results in vascular functionality tests^14–17^. Furthermore, experimental evidences from animal studies or in vitro models have shown that KL has protective effects against different forms of cardiovascular damage (vascular calcification, endothelial dysfunction or heart failure)^18–23^. One of the proposed mechanisms for the effects of KL on the vasculature is the modulation of the inflammatory response. Anti-inflammatory effects of KL already described include the reduction in the expression of pro-inflammatory cytokines^24^ and the increased secretion of anti-inflammatory factors^25,26^, the reduction of oxidative stress molecules^27,28^, the inhibition of the NFkB or NLRP3 inflammasome signaling pathways^29–32^, and the inhibition of the expression of endothelial adhesion molecules (ICAM1 and VCAM1)^33^. Furthermore, proinflammatory processes also have the ability to negatively modulate KL expression, thus shutting down a hypothetical feedback control system. KL is mainly expressed in the kidneys, but also in other tissues and cell types including monocytes, macrophages and lymphocytes^26,34,35^. In these cells, KL displays various anti-inflammatory effects^26,34–39^; therefore, the modulation of its production could represent a potential pathway for the regulation of the inflammatory response. However, there is a lack of knowledge about KL expression profile in PBCCs and its relationships with the inflammatory process during CVD. In this work, we aim to fill this gap by analyzing different markers of systemic and vascular inflammation and their relationships with *KL* gene expression in PBCCs and with serum levels of sKL in a group of patients with diagnosed atherosclerosis.

## Methods

### Patients and samples

This study follows a cross-sectional single center case–control design. Participants were consecutively enrolled from November 2014 to September 2017 in the Vascular Surgery and Transplant Coordination services of the University Hospital Nuestra Señora de Candelaria (UHNSC). The study protocol was approved by the UHNSC Ethics Committee and complied with ethical standards of the Declaration of Helsinki. Written informed consent was obtained from all participants.

Case group consisted in 76 patients older than 18 years which underwent an elective open vascular surgery procedure due to clinical atherosclerotic vascular disease. Exclusion criteria included hemodynamic instability during the surgical procedure (defined as a systolic blood pressure lower than 90 mmHg or the need for inotropes or vasopressors); history of chronic inflammatory, immunologic, or tumoral disease; positive serology to hepatitis B, hepatitis C, or HIV; acute inflammatory or infectious intercurrent episodes in the previous month; renal insufficiency, defined as an estimated glomerular filtration rate (eGFR) lower than 60 mL/min/1.73m^2^; institutionalization; receipt of immunotherapy or immunosuppressive treatment; and inability or unwillingness to provide informed consent. The distribution of clinical diagnosis was: 45 patients with PAD and intermittent claudication, 30 patients with transient ischemic attack (TIA), and 16 patients with abdominal aortic aneurysm (AAA). Two or more of these diagnoses were present in 14 patients. In all cases, the presence of established atherosclerotic vascular disease was confirmed with imaging studies that included computing tomography, magnetic resonance, and/or angiography procedures. During surgery, a sample of the carotid, aorta, femoral artery, or peripheral territories, according to the affected vessel, was obtained from the participants.

The control group consisted of 16 cadaveric organ donors, without any medical history or study indicating the presence of CVD. All organ donors underwent monitoring in the hospital prior to death. This was carried out by the Transplant Coordination Unit, which was responsible for the diagnosis of death, organs extraction, and vascular samples retrieval. Control subjects suffered brain death (10) or were controlled asystole donors (6). In order to minimize the inflammatory cascade occurring in the decease process, a corticosteroid treatment is routinely administered to donors (1 single bolus of methylprednisolone, 15 mg/kg) at the time of diagnosis of death (in cases of brain death) or before extubation (in asystole). Vascular samples were retrieved at the time of organ harvesting (4–6 h after time of death).

Whole blood samples (2.5 mL) were collected in either PAXgene Blood RNA tubes (BD, Franklin Lakes, NJ) and routine blood-collection tubes (BD serum separation transport tube—BD, Franklin Lakes, NJ). In case group, the collection was made at the time of surgery. In control subjects, samples were obtained at the moment of diagnosis (brain death) or while the patient was still alive (asystole). Serum fractions were isolated, aliquoted and immediately frozen at -80ºC. For methylation analysis, a blood-collection tube (BD K2EDTA tube—BD, Franklin Lakes, NJ) was drawn from 44 CVD patients and form 15 control subjects.

### Serum determinations

Routine biochemical and hematological parameters were determined using standardized tests at the UHNSC Clinical Analysis Service. Serum C-reactive protein (CRP) levels were measured using a highly sensitive automated immunoturbidimetric test on a Cobas 6000 analyzer (Roche Diagnostics GmbH), with a sensitivity of 0.3 mg/L and intra- and inter-assay coefficients of variation of 1.6 and 8.4%, respectively. Serum levels of the inflammatory cytokines TNFα and IL10 were measured using commercial ELISA assay kits (Human TNFα Quantikine HS ELISA HSTA00D and Human IL10 Quantikine D100B, R&D Systems) according to manufacturer's instructions. Samples with serum IL10 values below the kit's sensitivity limit (< 3.9 pg/mL) were reanalyzed with the commercial high sensitivity assay Human IL10 Quantikine HS HS100C (R&D Systems). Concentrations of serum sKL protein were measured by solid phase sandwich ELISA (human soluble α-Klotho assay kit, Immuno-Biological Laboratories) according to manufacturer’s instructions. The assay sensitivity for this kit was 6.15 pg/mL and the intra- and inter-assay coefficients of variation were 2.7–3.5% and 2.9–11.4%, respectively.

### RT-PCR and gene expression analysis

Vascular segments were completely homogenized in liquid nitrogen with a pestle and mortar. Total RNA was extracted using RNAzol RT according to manufacturer's instructions (Sigma Aldrich, MO, USA) and stored at − 80 °C. Total RNA from blood samples was isolated using PAXgene Blood RNA Kit (PreAnalytiX, Switzerland) following the manufacturer's guidelines. RNA was retrotranscribed to cDNA using a High Capacity RNA-to-cDNA kit (Applied Biosystems, CA, USA) for further use in quantitative RT-PCR (qRT-PCR).

*KL* gene cDNA was amplified by RT-PCR (KLcDNA-F:5'ACTCCCCCAGTCAGGTGGCG G3', KLcDNA-R:5'TGGGCCCGGGAAACCATTGCT3') to confirm its expression in PBCCs with the following conditions: 1.5 μL KAPA Taq Buffer B 10X, 0.75 μL MgCl_2_ 25 mM, 1.5 μL dNTPs 2 mM, 0.3 μL forward and reverse primer 20 μM, 0.06 μL KAPA Taq DNA pol 5U/μL and 2 μL DNA sample in a total volume of 15 μL. Thermal cycling conditions were 94 °C, 3 min; (94 °C, 30 s; 55 °C, 30 s; 72 °C, 30 s) × 35 cycles; 72 °C, 1 min. The result was confirmed by the presence of a 350 bp band in 1% agarose electrophoresis and sequencing.

Transcripts encoding for *KL, TNF, IL10, NFKB1*, *DNMT1, DNMT3A* and *GAPDH* genes were measured by TaqMan qRT-PCR with PerfeCTa FastMix II Low ROX (QuantaBio, MA, USA) in a 7500 Fast Real-Time PCR System (Applied Biosystems, CA, USA). TaqMan gene expression assays employed were: Hs00183100_m1 [*KL*], Hs00174128 m1 [*TNF*], Hs00961622_m1 [*IL10*], Hs00765730 m1 [*NFKB1*], Hs00945875_m1 [*DNMT1*], Hs00173377_m1 [*DNMT3A*] and Hs99999905_m1 [*GAPDH*] The level of target mRNA was estimated by relative quantification using the 2^-ΔΔCt^ method and *GAPDH* as housekeeping gene. Quantification of each cDNA sample was tested in triplicate.

### Promoter methylation analysis

DNA was extracted from PBCCs using the QIAamp DNA Blood Mini Kit (Qiagen, Hilden, Germany) according to the manufacturer's instructions. DNA quantity and purity were determined using a Nanodrop Lite Spectrophotometer (Thermo Fisher Scientific, MA, USA). 500–1000 ng of genomic DNA were subjected to conversion with sodium bisulfite using the EpiTect^®^ Fast DNA Bisulfite kit (QIAGEN) according to the supplier's instructions.

Posteriorly, *KL* gene promoter was amplified by methylation independent PCR (KL-MIP). Firstly, the − 1363 to + 74 region was pre-amplified (KL-MIPouterF: 5'GGGTAGGGAGGTAGGGATATTAG3’, KL-MIPouterR: 5'CCCAACAACACCAACAACAAC3’) and the region of interest (− 827 to − 258) was subsequently amplified using a 1/10–1/100 dilution of the previous PCR product as template (KL-MIPinnerF: 5’AATTTGGTGTTTGGTTTTTTAGGAG3’, KL-MIPinnerR: 5’CACCTATTTCTCCCAACTCCC3’). PCR reactions were performed with the KAPA2G Robust HotStart PCR kit (KAPA Biosystems), using the following conditions: 3 μL KAPA2G Buffer B 5X, 3 μL KAPA Enhancer 5X, 0.3 μL dNTPs 10 mM, 0.3 μL forward and reverse primer 20 μM, 0.12 μL KAPA2G Robust HotStart DNA pol 5U/μL and 2 μL DNA sample in a total volume of 15 μL. Thermal cycling conditions were 95 °C, 5 min; (95 °C, 30 s; 55 °C, 30 s; 72 °C, 90 s) × 25 cycles; 72 °C, 5 min for MIPouter, and 95 °C, 3 min; (95 °C, 15 s; 60 °C, 15 s; 72 °C, 15 s) × 35 cycles; 72 °C, 1 min for MIPinner. PCR products were sequenced and the methylation status of 11 CpG positions in the region between -648 and -560 was analyzed. Methylation levels were calculated as percentages: (number of methylated CpG positions/total number of CpG positions) × 100.

### Immunohistochemistry

Immunohistochemical and histological analyses of the vascular wall were performed in 37 patients and 10 controls. Sections of blood vessels were fixed in 4% buffered formalin for 24 h and subsequently dehydrated in ascending concentrations of ethanol, cleared in xylene and embedded in paraffin. Blocks were trimmed and 3 µm sections were processed for histology and immunohistochemistry. Hematoxylin and eosin (HE) and Masson's trichrome staining were performed. Primary antibodies used for immunohistochemistry were mouse monoclonal anti-IL10, 1:300 dilution (Santa Cruz Biotechnology Inc., Dallas, TX, USA) and rabbit monoclonal anti-TNFα, 1:100 dilution (Santa Cruz Biotechnology Inc.). For quantification analysis, a total of 5 images, that included intima and media layers, of each slide were captured and processed with a high-resolution video camera (Sony, DF-W-X710, Kōnan, Japan) connected to a light microscope (Nikon Eclipse 50i). Stained areas were quantified using ImageJ software (Rasband, W.S., ImageJ, National Institutes of Health, Bethesda, MD, USA). Results are expressed in square microns (µm^2^).

### Statistics

Continuous variables are reported as mean ± standard deviation (SD) or medians and interquartile ranges (IQR). Categorical data are presented as percentages. Continuous variables were assessed for normal distribution by D’Agostino-Pearson test, and those variables that presented non-normal distribution were log-transformed for statistical analysis. Differences among groups were analyzed by unpaired t test, Mann–Whitney test or one-way analysis of variance with Tukey's post hoc test. Categorical variables were compared between groups using Fisher's exact test. Correlation analysis was evaluated by Spearman correlation test. Multiple lineal regression analysis was performed using *KL* gene expression in PBCCs as dependent variable and eGFR, CRP, phosphorus, total cholesterol, vascular *TNF*/*IL10* ratio, PBCCs *TNF*/*IL10* ratio, serum TNFα/IL10 ratio and serum sKL levels were introduced as covariates. Pair correlations between these covariates and *KL* gene expression are shown in supplemental material. Multiple logistic regression analyses were performed to assess independent predictors of the presence of CVD and of atherosclerotic plaque. For this purpose, we adopted three models: in model 1, we introduced conventional risk factors as covariates (age, sex, smoking, hypertension (HT), diabetes mellitus (DM)); in model 2, we additionally included *TNF*/*IL10* gene expression ratio in PBCCs and serum TNFα/IL10 ratio; in model 3, we adjusted the analysis by including *KL* gene expression in PBCCs and sKL serum concentrations. Values of *P* < 0.05 were considered significant. Statistical analyses were performed using IBM SPSS Statistics V.19 (IBM Corporation, NY, USA) and GraphPad Prism 6.01 software (GraphPad Software, CA, USA).

## Results

### Clinical data

The group of CVD patients tended to a lower body mass index (27.5 ± 3.6 vs. 29.4 ± 3.2, *P* = 0.06), a higher prevalence of HT (77.6% vs. 56.3%, *P* = 0.11), with no differences in the frequency of DM (43.4% vs. 31.3%, *P* = 0.42). Regarding laboratory data, CVD patients presented lower serum LDL (88.2 ± 37.2 vs. 121.0 ± 26.1, *P* < 0.01) and calcium (9.1 ± 0.5 vs. 9.3 ± 0.9, *P* < 0.05) (Table 1). The use of antiplatelet agents (91% vs. 16.7%, *P* < 0.0001), angiotensin-converting enzyme inhibitors or angiotensin receptor antagonists (51.3% vs. 25.0%, *P* < 0.05) and statins (85.5% vs. 8.3%, *P* < 0.0001) was significantly higher in the CVD group (Table 1).

**Table 1:** Clinical characteristics and biochemical assessments of the patients included in the study.

	CVD (n = 76)	Non CVD (n = 16)	*P* value
Age (years)	65.3 ± 7.4	63.2 ± 9.3	0.38
Sex (M/F)	59 / 17	10/6	0.22
Smoker (%)	77.6	75.0	0.75
BMI (kg/m^2^)	27.5 ± 3.6	29.4 ± 3.2	0.06
HT (%)	77.6	56.3	0.11
DM (%)	43.4	31.3	0.42
**Pharmacological treatment**			
Antiaggregants (%)	91.0	16.7	< 0.0001
Beta-blockers (%)	27.6	16.7	0.34
ACEI/ARA2 (%)	51.3	25.0	< 0.05
CCB (%)	25.0	16.7	0.35
Statins (%)	85.5	8.3	< 0.0001
**Laboratory data**			
eGFR (mL/min/1.73 m^2^)	89.6 ± 13.2	89.8 ± 26.7	0.61
Creatinine (mg/dL)	0.84 ± 0.2	0.93 ± 0.5	0.48
Albumin (g/dL)	3.8 ± 0.5	3.9 ± 0.6	0.43
Calcium (mg/dL)	9.1 ± 0.5	9.3 ± 0.9	< 0.05
Phosphorus (mg/dL)	3.6 ± 0.5	3.5 ± 0.9	0.67
Uric acid (mg/dL)	5.9 ± 1.4	6.1 ± 1.5	0.63
Glucose (mg/dL)	117.3 ± 37.1	131.2 ± 39.4	0.21
Cholesterol (mg/dL)	165.4 ± 48.7	190.2 ± 29.9	0.12
HDL (mg/mL)	43.2 ± 11.2	47.5 ± 14.6	0.67
LDL (mg/dL)	88.2 ± 37.2	121.0 ± 26.1	< 0.01
Neutrophils (/mL)	7494 ± 5081	8287 ± 5815	0.50
Lymphocytes (/mL)	2020 ± 998.2	1894 ± 729.9	0.91
**Serum inflammatory markers**			
NLR	3.8 ± 1.9	2.8 ± 1.2	0.21
CRP (mg/L)	2.9 ± 2.6	1.9 ± 0.9	0.38
TNFα (pg/mL)	1.04 (0.80–1.44)	1.37 (0.90–1.98)	0.11
IL10 (pg/mL)	3.93 (0.61–10.13)	10.38 (7.52–30.20)	< 0.001
TNFα/IL10	0.28 (0.10–1.62)	0.11 (0.02–0.27)	< 0.01
sKL (pg/mL)	507.7 (361.4–656.6)	1007 (590.4–1883)	< 0.01

*ACEI/ARA2* Angiotensin converting enzyme inhibitor/angiotensin receptor antagonist 2; *BMI* Body mass index; *CCB* Calcium channels blockers; *CRP* C-reactive protein; *DM* Diabetes mellitus; *eGFR* Estimated glomerular filtration rate; *HDL* High-density lipoprotein; *HT* hypertension; *IL10* Interleukin 10; *LDL* Low-density lipoprotein; *NLR* Neutrophil-to-lymphocyte ratio; *TNFα* Tumor necrosis factor alpha.

### Inflammatory markers in serum, PBCCs and vascular wall

We evaluated systemic inflammation by determining serum levels of the cytokines TNFα (pro-inflammatory) and IL10 (anti-inflammatory), and also of CRP and the neutrophil-to-lymphocyte ratio (NLR). The CVD group showed no significant differences for the systemic levels of TNFα [1.04 (0.80 to 1.41) vs. 1.37 (0.90 to 1.98); *P* = 0.11], while IL10 concentrations were reduced compared to the control group [3.93 (0.61 to 10.13) versus 10.38 (7.52 to 30.20), *P* < 0.001]. In order to assess the global inflammatory status, values for the TNFα/IL10 ratio were also calculated for each sample. This parameter was significantly higher in the CVD group [0.28 (0.10 to 1.62) vs. 0.11 (0.02 to 0.27), *P* < 0.01]. CRP levels (3.8 ± 1.9 vs. 2.8 ± 1.2; *P* < 0.21) and the NLR (2.9 ± 2.6 vs. 1.9 ± 0.9; *P* < 0.38) were higher in the CVD group but differences did not reach statistical significance. Serum sKL levels were significantly diminished in the CVD group [507.7 (361.4 to 656.6) vs. 1007 (590.4 to 1883), *P* < 0.01] (Table 1).

**Figure 1:**
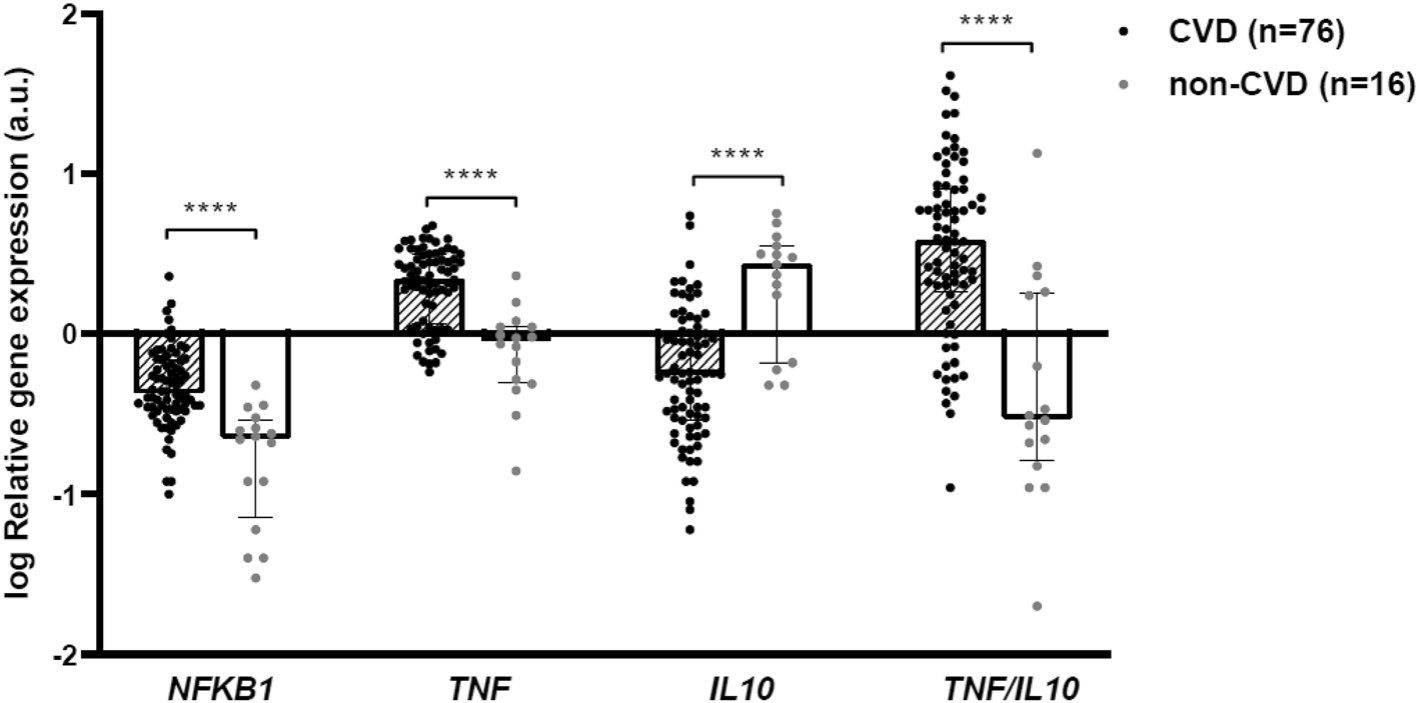
Inflammatory markers in PBCCs of CVD and non-CVD subjects. Relative gene expression levels of *NFKB1, TNF* and *IL10 loci* and their ratio. a.u.: arbitrary units. Bars and range represent median and IQR. ***: *P* < 0.001; ****: *P* < 0.0001.

Inflammatory markers were also investigated in PBCCs. Gene expression levels of *NFKB1*, which codifies for the nuclear factor-kappa-B (NFκB) p105 subunit implied in the pro-inflammatory NFκB pathway, were significantly higher in PBCCs of the CVD group [log RQ: − 0.37 (− 0.47 to − 0.17) versus − 0.65 (− 1.15 to − 0.54), *P* < 0.0001]. Similarly, *TNF* gene expression levels were significantly higher in PBCCs of the CVD group [log RQ: 0.34 (0.06 to 0.49) versus − 0.05 (− 0.30 to 0.04), *P* < 0.0001], while transcript levels of *IL10* gene were higher in the control group [log RQ: − 0.25 (− 0.53 to 0.01) vs. 0.43 (− 0.18 to 0.55), *P* < 0.0001]. Thus, the *TNF*/*IL10* expression ratio presented significantly higher values in the CVD group compared to control individuals [log: 0.58 (0.26 to 0.90) versus − 0.52 (− 0.79 to 0.26), *P* < 0.0001] (Fig. 1).

**Figure 2:**
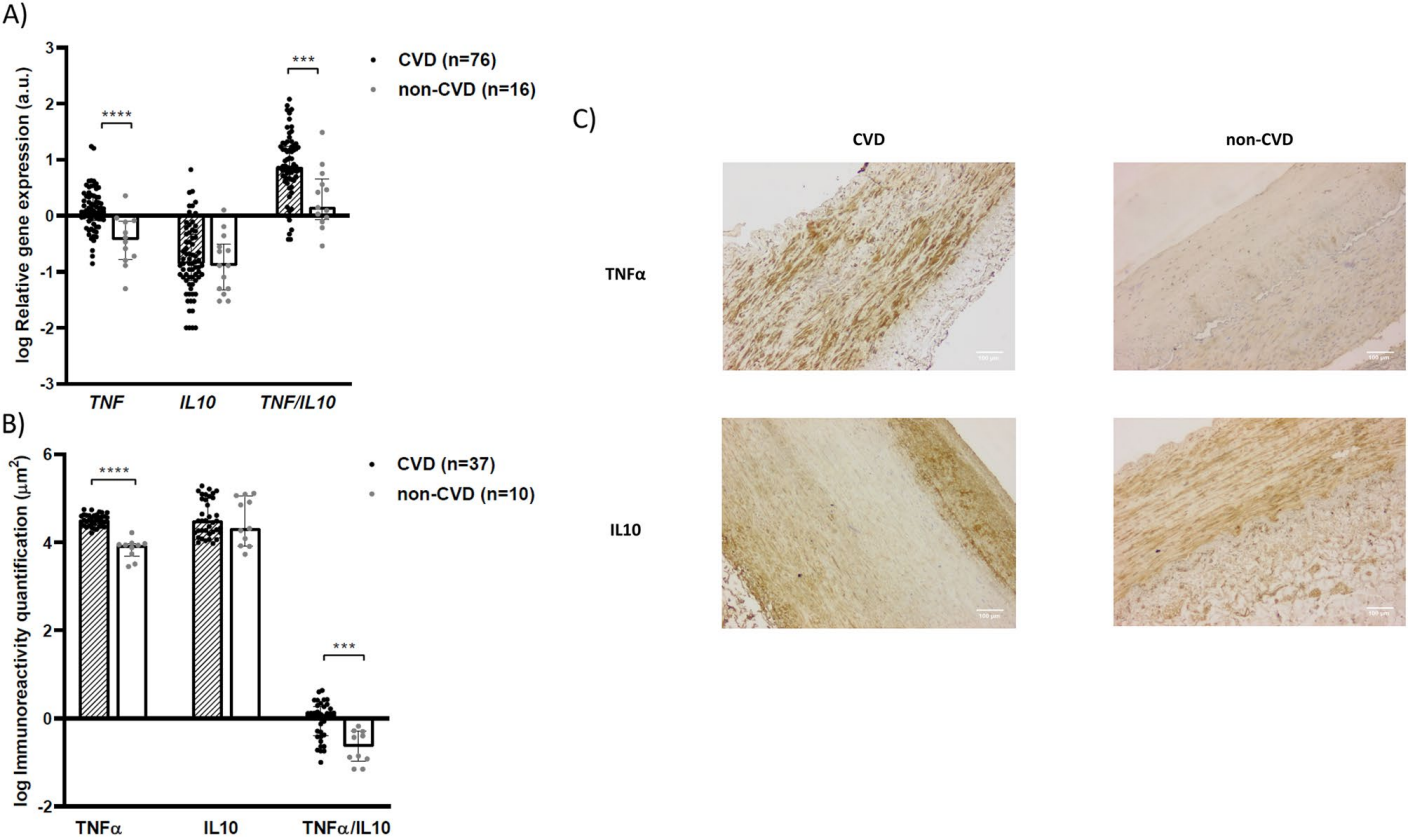
Inflammatory markers in the vascular wall of CVD and non-CVD subjects. (**A**) Relative gene expression levels and (**B**) immunoreactivity levels for TNF, IL10 and their ratio. (**C**) Immunohistochemistry images for TNFα and IL10. a.u.: arbitrary units. Bars and range represent median and IQR. ***: *P* < 0.001; ****: *P* < 0.0001.

Vascular gene expression analysis revealed that patients with CVD had higher expression levels of *TNF* [log RQ: 0.09 (− 0.08 to 0.34) versus − 0.43 (− 0.78 to − 0.09), *P* < 0.0001] with no differences for *IL10* expression [log RQ: − 0.85 (− 1.16 to − 0.33) versus − 0.89 (− 1.33 to -0.50), *P* = 0.64]. A more pronounced pro-inflammatory state was observed in patients with CVD compared to non-CVD subjects when assessing the *TNF/IL10* ratio [log: 0.87 (0.64 to 1.24) versus 0.15 (− 0.07 to 0.66), *P* < 0.001] (Fig. 2A). Inmmunohistochemical analysis (Fig. 2C) revealed higher vascular immunoreactivity levels for TNFα protein in patients with established CVD compared to control subjects [log μm^2^: 4.51 (4.38 to 4.61) versus 3.94 (3.68 to 3.97), *P* < 0.0001], while immunoreactivity levels for IL10 were similar in both groups [log μm^2^: 4.49 (4.22 to 5.00) versus 4.32 (3.92 to 5.07), *P* = 0.66]. Values for the TNFα/IL10 ratio were also significantly higher in CVD patients compared to control group [log: 0.09 (− 0.40 to 0.28) versus − 0.64 (− 0.98 to − 0.30), *P* < 0.001] (Fig. 2B).

### *KL* expression and epigenetic regulation in PBCC

**Figure 3:**
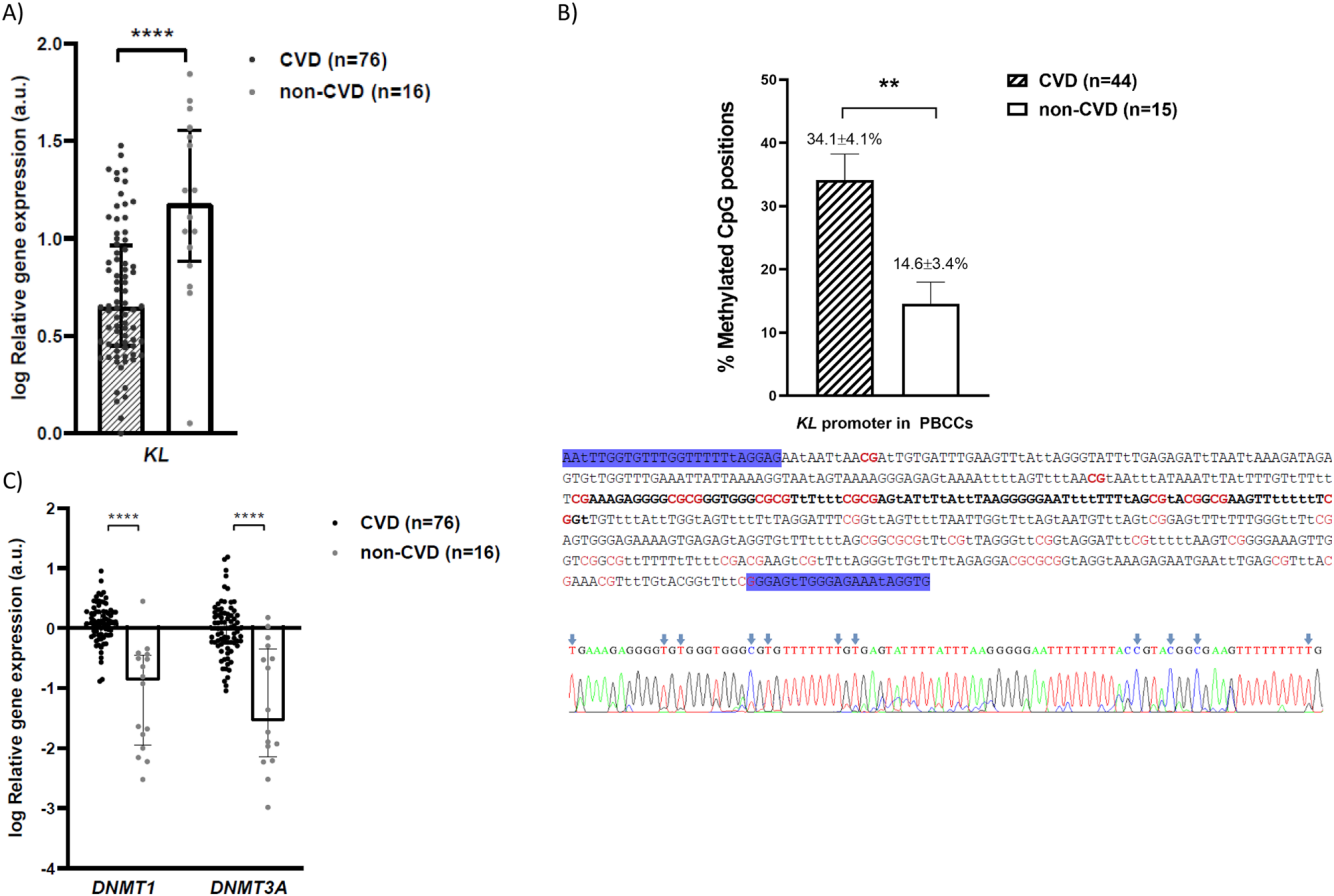
KL gene expression and epigenetic regulation in PBCCs of CVD and non-CVD subjects. (**A**) Relative gene expression levels of *KL locus*, (**B**) methylation levels of 11 CpG positions (red) in the − 648 and − 560 regions (bold) of *KL* gene promoter (lowercase “t” represents unmethylated cytosines after conversion with sodium bisulfite), (**C**) relative gene expression levels of *DNMT1* and *DNMT3A loci*. a.u.: arbitrary units. Bars and range represent median and IQR (**A** and **C**) or mean and SEM (**B**). **: *P* < 0.01; ****: *P* < 0.0001.

Quantitative analysis of *KL* gene expression in PBCCs showed that patients with established CVD presented significantly lower levels of the mRNA compared to control subjects [log RQ: 0.65 (0.45 to 0.97) versus 1.18 (0.88 to 1.57), *P* < 0.0001; a reduction of 56.4%] (Fig. 3A). We did not observe significant differences in *KL* gene expression in PBCCs when we stratified the patients according to their co-morbidities (Supplementary Fig. S1). *KL* gene promoter has two CpG islands between − 848 and + 88 positions, and their hypermethylation is associated with gene expression downregulation. We investigated the methylation status of 11 positions in the promoter region (between − 648 and − 560 positions) of the *KL* gene promoter in PBCCs from the study subjects. The analysis showed that the group of CVD patients had a higher degree of methylation of the interrogated positions compared to the control group (34.1 ± 4.1% vs. 14.6 ± 3.4%; *P* < 0.01) (Fig. 3B). Moreover, this same group of patients presented higher expression levels of two genes codifying for DNA-methyltransferases responsible for genome methylation: *DNMT1* [log RQ: 0,10 (− 0.11 to 0.28) versus − 0.86 (− 1.94 to − 0.45); *P* < 0,0001] and *DNMT3A* [log RQ: − 0.03 (− 0.26 to 0.24) versus − 1.54 (− 2.15 to − 0.35); *P* < 0.0001] (Fig. 3C).

### Associations between inflammatory markers and *KL* expression in PBCCs and serum sKL

We developed correlation analyses to study the bivariate associations of different vascular, PBCCs and serum inflammatory parameters with *KL* expression in PBCCs and sKL serum concentrations (Table 2). Pro-inflammatory markers in PBCCs were significantly and inversely associated with the gene expression of *KL* in these cells (*NFKB1*: r = − 0.281, *P* < 0.01; *TNF:* r = − 0.310, *P* < 0.01; *TNF*/*IL10* ratio: r = − 0.509, *P* < 0.0001), while *IL10* expression levels was directly correlated (r = 0.500, *P* < 0.0001). Regarding serum inflammatory parameters, we only found a significant direct association for IL10 circulating levels and *KL* expression in PBBCs (r = 0.253, *P* < 0.05). Interestingly, vascular *TNF*/*IL10* ratio presented a significant inverse correlation with *KL* expression in PBCCs (r = − 0.337, *P* < 0.01). *KL* and *DNMT1* expressions were significantly and inversely correlated (r = − 0.257, *P* < 0.05), while association between *KL* and *DNMT3A* expressions did not reach statistical significance (r = − 0.166, *P* = 0.12). Curiously, pro-inflammatory markers in PBCCs were directly associated with both *DNMT1* and *DNMT3A*, while *IL10* expression in these cells was inversely correlated with both genes encoding for DNA-methyltransferases (Table 2). Serum levels of sKL presented significant correlations with inflammatory markers in PBCCs, being direct with pro-inflammatory markers (*TNF*: r = − 0.295, *P* < 0.01; r = − 0.294, *P* < 0.01 for *TNF*/*IL10*) and inverse with IL10 gene expression (IL10: r = 0.234, *P* < 0.05). We also observed that circulating levels of sKL tended to be positively associated with *KL* expression in these cells (r = 0.173, P = 0.11), but it did not present significant associations with vascular markers of inflammation (Table 2).

**Table 2:** Bivariate correlation analysis between PBCCs expression, vascular tissue expression and serum levels parameters.

	PBCCs expression (n = 92)					
	*KL* (a.u.)		*DNMT1* (a.u.)		*DNMT3A* (a.u.)	
	r	*P* value	r	*P* value	r	*P* value
**PBCCs expression**						
*KL* (a.u.)	1.000					
*DNMT1* (a.u.)	− 0.257*	< 0.05	1.000			
*DNMT3A* (a.u.)	− 0.166	0.12	0.692*	< 0.0001	1.000	
*NFKB1* (a.u.)	− 0.281*	< 0.01	0.580*	< 0.0001	0.484*	< 0.0001
*TNF* (a.u.)	− 0.310*	< 0.01	0.487*	< 0.0001	0.308*	< 0.01
*IL10* (a.u.)	0.500*	< 0.0001	− 0.253*	< 0.05	− 0.235*	< 0.05
*TNF*/*IL10*	− 0.509*	< 0.0001	0.397*	< 0.001	0.306*	< 0.01
**Serum levels**						
sKL (pg/mL)	0.173	0.11	− 0.186	0.08	− 0.041	0.70
TNFα (pg/mL)	0.162	0.12	− 0.198	0.16	0.283*	< 0.01
IL10 (pg/mL)	0.253*	< 0.05	− 0.403*	< 0.0001	− 0.155	0.14
TNFα/IL10	− 0.186	0.08	0.334	< 0.01	0.069	0.52

	Vascular tissue expression (n = 92)					
	*TNF* (a.u.)		*IL10* (a.u.)		*TNF*/*IL10*	
	r	*P* value	r	*P* value	r	*P* value
**PBCCs expression**						
*KL* (a.u.)	− 0.168	0.13	0.159	0.15	− 0.337*	< 0.01
*DNMT1* (a.u.)	0.353*	< 0.01	0.069	0.53	0.236*	< 0.05
*DNMT3A* (a.u.)	0.309*	< 0.01	0.104	0.35	0.184	0.10
*NFKB1* (a.u.)	0.379*	< 0.001	− 0.006	0.95	0.302*	< 0.01
*TNF* (a.u.)	0.275*	< 0.05	0.037	0.74	0.196	0.08
*IL10* (a.u.)	− 0.061	0.58	0.143	0.19	− 0.270*	< 0.05
*TNF*/*IL10*	0.166	0.13	− 0.125	0.26	0.311*	< 0.01
**Serum levels**						
sKL (pg/mL)	− 0.179	0.11	− 0.078	0.49	− 0.070	0.54
TNFα (pg/mL)	− 0.185	0.10	− 0.021	0.85	− 0.144	0.20
IL10 (pg/mL)	− 0.244*	< 0.05	0.137	0.22	− 0.302*	< 0.01
TNFα/IL10	0.166	0.14	− 0.137	0.22	0.233*	< 0.05

We performed a multiple regression analysis using *KL* gene expression in PBCCs as the dependent variable to analyze how different clinical and inflammatory parameters are able to predict its levels. The eGFR, CRP, phosphorus, and cholesterol serum levels, vascular and PBCC gene expression ratio *TNF*/*IL10*, serum TNFα/IL10 ratio and sKL levels were used as covariates (Table 3). The analysis showed that *TNF*/*IL10* ratio in PBCCs (β = − 0.234, *P* < 0.05) and systemic sKL concentrations (β = 0.282, *P* < 0.05) were significantly associated with *KL* expression in circulating cells (adjusted R^2^ = 0.287, *P* < 0.01).

**Table 3:** Multiple regression analysis for *KL* expression in PBCCs as dependent variable.

	PBCCs *KL* expression (n = 92)				
	Adjusted R^2^	Standarized β	t	Tolerance	*P* value
Model	0.287				< 0.01
eGFR (mL/min/1.73 m^2^)		0.178	10.58	0.96	0.12
CRP (mg/L)		− 0.153	− 10.40	0.88	0.17
Phosphorus (mg/dL)		0.019	0.17	0.95	0.86
Cholesterol (mg/dL)		0.145	10.32	0.94	0.19
Vascular *TNF*/*IL10*		0.148	10.36	0.96	0.18
PBCCs *TNF*/*IL10*		− 0.234	− 20.08	0.90	< 0.05
Serum TNFα/IL10		0.024	0.22	0.89	0.83
Serum sKL (pg/mL)		0.282	20.46	0.86	< 0.05

### *KL* expression in PBCCs and serum sKL levels according to the presence of developed atherosclerotic damage

**Figure 4:**
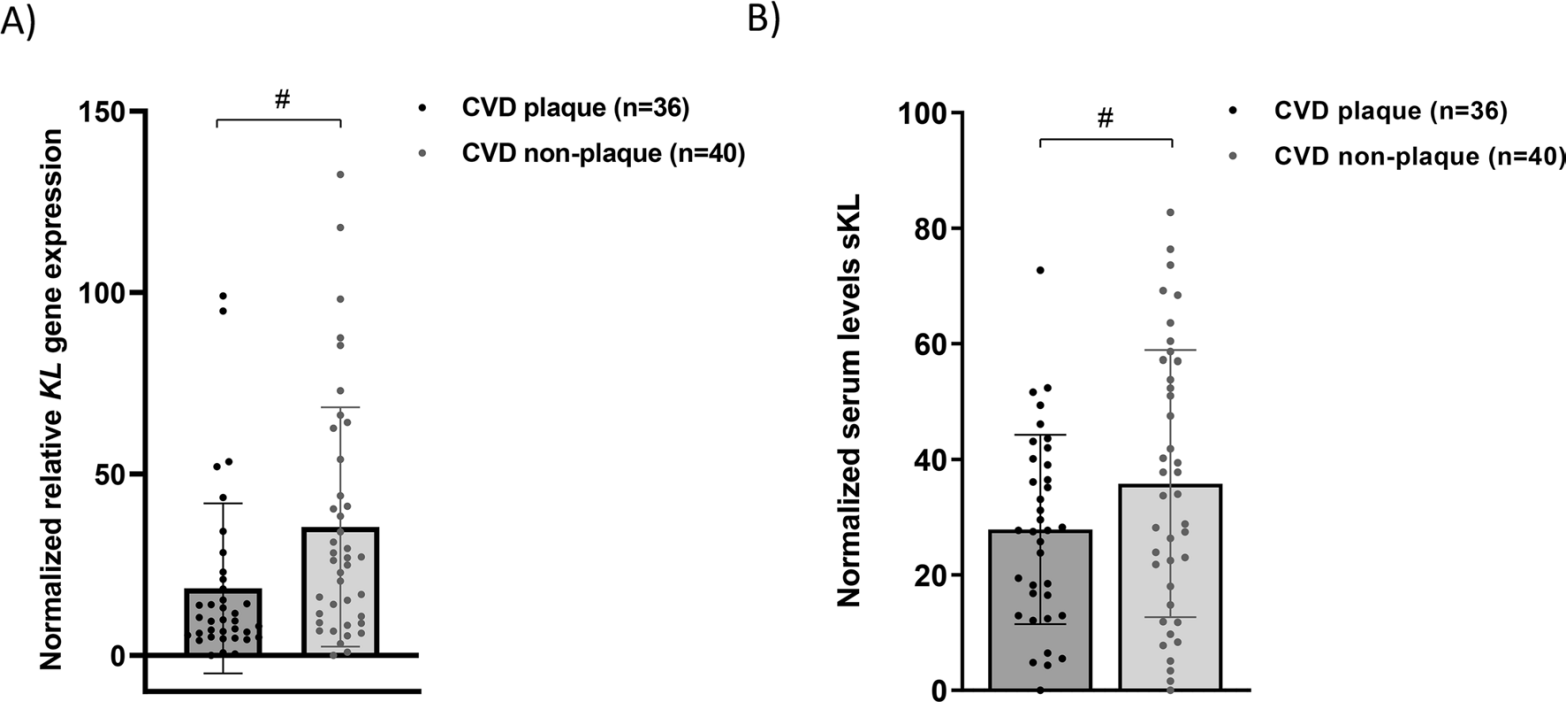
KL levels according to the presence of developed atherosclerotic plaque in CVD patients. (**A**) Relative gene expression levels of *KL locus* in PBCCs, (**B**) Serum levels of sKL (pg/mL). a.u.: arbitrary units. Bars and range represent median and IQR. **: *P* < 0.01; ****: *P* < 0.0001 vs non-CVD group; #: *P* < 0.05.

In the CVD group, 36 patients showed a developed atherosclerotic plaque (identifiable by macroscopic visualization) in the vascular tissue samples, while 40 showed no obvious signs of vascular damage. After normalizing the data against the non-CVD group (mean value for the different variables of this group was considered as 100%), we stratified the patients in the CVD group according to the presence of these plaques. We found that *KL* gene expression levels in PBCCs were significantly lower in those individuals with vascular damage [Normalized RQ: 9.66 (5.18 to 20.33) versus 26.53 (9.46 to 51.56), *P* < 0.05]. Similarly, serum sKL concentrations in patients with developed atherosclerotic plaque in the tissue sample were significantly lower [Normalized serum levels: 27.87 ± 16.43 versus 35.78 ± 23.10, *P* < 0.05] (Fig. 4).

To study the association of these KL elements with the presence or absence of CVD and of atherosclerotic damage, we developed multiple logistic regression analyses with iterative models that included classic cardiovascular risk factors (model 1), PBCCs and serum inflammatory markers (model 2), and *KL* expression in leukocytes and serum sKL levels (model 3). In a final adjusted model, we observed that expression in PBCCs of *KL* gene and serum sKL concentration were protective factors for the presence of both CVD and developed atherosclerotic plaque (Table 4, Supplementary Fig. S3).

**Table 4:** Logistic regression analysis for presence of CVD or presence of atherosclerotic plaque.

	Presence of CVD (n = 92)		Presence of atherosclerotic plaque (n = 92)	
	OR (CI 95%)	*P* value	OR (CI 95%)	*P* value
**Model 1**				
Age	1.04 (0.94–1.15)	0.47	1.01 (0.95–1.08)	0.72
Sex	0.44 (0.12–1.68)	0.23	0.70 (0.24–2.04)	0.52
Smoking	1.21 (0.23–6.26)	0.82	1.82 (0.56–5.88)	0.32
HT	0.96 (0.21–4.28)	0.95	0.76 (0.26–2.18)	0.60
DM	1.33 (0.35–5.02)	0.67	1.21 (0.50–2.95)	0.68
**Model 2**				
Age	1.06 (0.95–1.18)	0.31	1.01 (0.95–1.08)	0.79
Sex	0.29 (0.05–1.51)	0.14	0.78 (0.26–2.39)	0.67
Smoking	2.42 (0.27–21.46)	0.43	1.78 (0.53–5.96)	0.35
HT	2.85 (0.46–17.54)	0.26	0.72 (0.24–2.11)	0.53
DM	0.78 (0.17–3.57)	0.75	1.40 (0.55–3.56)	0.48
PBCCs *TNF*/*IL10*	1.44 (0.98–2.11)	0.06	0.99 (0.93–1.06)	0.78
Serum TNFα/IL10	3.29 (0.52–20.89)	0.21	1.12 (0.95–1.32)	0.17
**Model 3**				
Age	1.16 (0.96–1.40)	0.12	1.03 (0.96–1.11)	0.39
Sex	0.12 (0.01–1.30)	0.08	0.60 (0.18–2.03)	0.41
Smoking	5.06 (0.21–120.38)	0.32	2.01 (0.54–7.45)	0.30
HT	4.65 (0.42–51.31)	0.21	0.84 (0.24–2.93)	0.78
DM	0.36 (0.03–4.08)	0.41	1.14 (0.40–3.25)	0.80
PBCCs *TNF*/*IL10*	1.09 (0.79–1.51)	0.62	0.95 (0.88–1.02)	0.16
Serum TNFα/IL10	1.87 (0.33–10.63)	0.48	1.10 (0.93–1.31)	0.25
PBCCs *KL*	0.86 (0.75–0.97)	< 0.05	0.89 (0.81–0.99)	< 0.05
Serum sKL	0.99 (0.98–0.99)	< 0.05	0.99 (0.98–0.99)	< 0.05

## Discussion

Circulating monocytes, macrophages, and lymphocytes play a central role in the development of the atherosclerotic lesion. Although endothelial dysfunction is the main prerequisite to unleash the atherogenic process, activation and adhesion of these PBCCs to the vascular wall are the starting point. In addition, these circulating cells are mainly responsible for the resolution of the inflammatory response at local level in the vasculature, establishing a balance between pro-inflammatory stimuli and inflammation-resolving processes. In atherosclerotic plaques we can find subpopulations of these cells that will produce pro-inflammatory mediators with functions of chemotaxis, infiltration and proliferation of monocytes/macrophages in the damaged area, development of foam cells by accumulation of LDLox in macrophages^40^, phenotypic modulation of resident vascular cells or destabilization of the plaque and thrombogenesis^41^. Beyond the vascular wall, these circulating immune cells contribute to the systemic meta-inflammatory state that usually accompanies CVD which is originated in the adipose tissue as a response to metabolic alterations associated with cardiovascular risk factors (CVRF)^42,43^. KL is an anti-aging factor that can be found as a transmembrane glycoprotein or as a soluble factor (sKL) produced by shedding of the first one or directly secreted by cells (this one correspond to a protein produced from an alternative splicing transcript of *KL* gene)^44^. This factor is mainly expressed in the kidneys, parathyroid glands or brain, and to a lesser extent in reproductive organs, skeletal muscle or vasculature^16,45,46^. In humans, the involvement of KL deficiency in various pathological processes associated to aging has been stated, such as chronic kidney disease (CKD)^9^, cancer^10^, CVD^12^, or defective angiogenesis in scleroderma disease^13^. Several mechanisms have been described for the role of KL in these diseases, one of interest being its various anti-inflammatory activities^24–26,29–33^. The ability of pro-inflammatory mediators to repress KL expression suggests a relationship where the balance between both elements may affect the outcome of the inflammatory response^45,47–49^. Regarding CVD, multiple studies have shown that KL develop protective effects in the vasculature, being able to prevent vascular calcification^18,19^, endothelial dysfunction^20–22^ or heart failure^23^. Gene expression of this factor has been observed in different PBCCs, such as monocytes, macrophages^26,35–39^ and CD4 + lymphocytes^34^. Its presence in these cells should also be considered, albeit indirectly, as a possible actor involved in the preservation of vascular function.

In our study we showed that *KL* gene expression in PBCCs is reduced in patients with clinical diagnosis of atherosclerotic vascular disease in relation to healthy subjects. Previous studies have stated that different forms of CVD are associated with deficiency of KL expression in the vasculature^12,45^ or circulating levels of sKL^11,12,14,15,17^. These results would extend the range of tissues/cells whose expression of this factor is compromised during CVD to the PBCCs. Although a reasonable proportion of CVD patients had other co-morbidities (HT, 77.6% and DM, 43.4%), no differences were observed in *KL* expression levels in PBCCs between patients who had these and those who did not (Supplementary Fig. S1). One of the epigenetic mechanisms that regulates *KL* gene expression is hypermethylation of the promoter in the − 1200 bp region upstream^50^. This region is extremely rich in GC (65.9% G + C), with two CpG islands located between positions − 848 and + 88^51^. Methylation of this promoter is responsible for *KL* predominant expression in the kidney compared to other tissues, and it has been associated with decreased expression levels in the kidney and peripheral blood mononuclear cells during CKD^51–53^. Furthermore, different pharmacological approaches have shown the ability to recover *KL* expression through demethylation of its promoter^54,55^. In our study, analysis of the methylation status of *KL* gene promoter in PBCCs revealed a higher methylation degree of the interrogated CpG positions in the CVD group. Moreover, we observed that this same group presented higher expression levels of *DNMT1* and *DNMT3A* genes (which encode for DNA-methyltransferases implicated in genome methylation). Besides, we detected an inverse significant correlation between *KL* expression and *DNMT1* expression. All these observations would suggest that this epigenetic mechanism is likely to modulate the observed *KL* downregulation in PBCCs during CVD. To our knowledge, this is the first work in which *KL* expression and methylation of its promoter has been assessed in blood circulating cells in the context of atherosclerotic vascular disease.

The expression of KL by different blood-circulating cells has been related to repression of the inflammatory response induced by lipopolysaccharide through proteolysis of TLR4 in the plasmatic membrane^37^, suppression of the stress response of the Golgi apparatus and endoplasmic reticulum, reduction of oxidative stress and pro-inflammatory cytokines, as well as to increased production of anti-inflammatory cytokines and preservation of immune function^26,38^. It also participates in the polarization of macrophages towards an M2 anti-inflammatory phenotype^39^. The exogenous protein has also been shown to have a modulating effect on circulating blood cells through repression of pro-inflammatory cytokines secretion^36,56^. All these mechanisms exerted by leukocytes play key roles in the atherosclerotic process and, therefore, make *KL* expression in these cells an interesting target in such scenario. Although it is necessary to study in depth what are the consequences of the loss of KL in these blood-circulating cells, it is presumable to hypothesize that a lack of its anti-inflammatory activity would play some role in the progression of vascular damage (at least, it would be expected that leukocyte downregulation of *KL* gene may predispose to decompensation of the inflammatory response in a pro-inflammatory sense).

In this study we found that *KL* expression in PBCCs was inversely associated with markers of pro-inflammatory response in these same cells, while it presented a direct correlation with the expression of the anti-inflammatory gene *IL10*. Interestingly, *DNMT1* and *DNMT3A* gene expression were also associated with all these inflammatory markers, but in an opposite direction (directly with pro-inflammatory markers and inversely with anti-inflammatory). Moreover, we observed similar associations between the expression in PBCCs of *KL*, *DNMT1* and *DNMT3A* genes and parameters of systemic inflammation (serum concentrations of TNFα, IL10 and their ratio). Together, these results highlight the existence of a negative relationship between the inflammatory process and the expression of *KL* in PBCCs. Other studies have appreciated similar associations between the development of diseases characterized by a pro-inflammatory profile and the reduction of *KL* gene expression in these cells^34,36,57^. Our observations here support such findings in the frame of CVD. In addition, our results also suggest that the increase in the pro-inflammatory systemic response would be associated with a greater presence of the elements responsible for DNA methylation and, therefore, with higher methylation processes.. According with this, previous reports have already observed that low-grade systemic inflammation is related to changes in DNA methylation patterns implicated in macrophage polarization towards pro-inflammatory phenotypes^58^ or with cardiometabolic phenotypes^59^. In vitro and in vivo experimental models have clearly stated that pro-inflammatory mediators (such as TNFα, TWEAK, or IFNγ among others) can repress KL expression^45,47–49^. Uremic toxins also have the ability to decrease KL expression, and it has been experimentally shown to occur through hypermethylation of its promoter^53^. Although to the best of our knowledge, it has not yet been demonstrated that this is the mechanism by which pro-inflammatory cytokines are capable to downregulate *KL* expression, it is plausible to assume that this is possible. In this paper, we only point to the existence of links between such processes, but taken together, our results could suggest that negative modulation of *KL* expression in PBCCs by the CVD pro-inflammatory environment might occur by hypermethylation of its promoter. However, experimental in vitro validation in leukocytes is necessary to determine if pro-inflammatory cytokines have such direct effect on *KL* promoter.

Regarding the vascular wall, we also observed that higher values of the *TNF*/*IL10* ratio are inversely associated with *KL* expression in PBCCs and, again, directly related to DNA-methyltransferases *loci* expression (only significant for *DNMT1*). This might indicate that a loss of *KL* expression in leukocytes is also linked to the inflammatory state of the vessel, which would extend the role of leukocyte KL in the modulation of athero-inflammation In the systemic circulation, sKL protein is the form mainly associated with its vasculoprotective effects. Different studies have linked decreased serum or plasma concentrations of sKL with the appearance of various forms of CVD^12–17^. In the present study, we have analysed how circulating sKL concentrations are associated with the inflammatory response in PBCCs or in the vascular wall. We found only significant associations that point to a negative relationship between sKL levels and the pro-inflammatory response in leukocytes. These results provide another point of connection between the inflammatory phenomenon underlying CVD and this anti-aging molecule.

We also showed that during CVD occurs a reduction of *KL* gene expression in circulating leukocytes. To assess this, we analysed *KL* expression levels in PBCCs and sKL serum levels according to the presence or absence of atherosclerotic plaque in an advanced stage of development (macroscopically visible in the vascular fragments). We observed that patients with advanced vascular lesion presented lower levels of expression for this gene in PBCCs and serum concentration of the soluble protein compared to those without observable plaque. Furthermore, in a multiple logistic regression model for the presence of atherosclerotic plaque as dependent variable, which included classical CVRF and inflammatory parameters as predictors, we observed that only *KL* gene expression in PBCCs or serum sKL levels acted as protective factors. Altogether, these results would indicate that the systemic KL (either the serum protein or the factor produced by leukocytes) might play a role in the development of the atherosclerotic lesion. The appearance of atherosclerotic plaques in specific regions of the vasculature (such as carotid or aorta) is directly related to the total atherosclerotic burden in the entire vascular tree^60^. Thus, it is possible to broadly assume that the observation of atheromatous plaques in our vascular tissue samples recovered from the surgeries of the CVD group implies a high degree of atherosclerotic burden in the patient. However, we are aware that this approach is not the most accurate to assess this parameter.

We realize that the present study has a series of limitations that must be taken into account when considering its conclusions, including: (1) small sample size (as a consequence, the ability to describe certain associations is limited), (2) nature of the control group (since this group consists of cadaveric organ donors and samples were retrieved in the early stages after death, it is presumable to consider that some inflammatory targets of the study may be affected by the decease process. and therefore, future studies are needed in living subjects free of CVD to validate our results), (3) impossibility to infer causality (this is a cross-sectional observational study that only allows to detect associations between the analysed variables, not their causes. Such associations need to be validated in experimental models), (4) influence of confounding variables not considered (it is plausible the existence of potential confounding factors not considered that could influence the observed associations), (5) lack of measurement of other inflammatory parameters (systemic and local inflammatory response in atherosclerosis involves other inflammatory mediators with significant roles in the process, such as IL1β, IL6, or TGFβ), (6) lack of measurement of mineral metabolism parameters (KL plays a relevant role in calcium and phosphorus metabolism, interacting with other molecules with a potential impact on CVD, such as FGF23 or vitamin D) and (7) limited assessment of the degree of methylation of *KL* gene promoter (this regulatory region includes two large CpG islands in a space of 936 bp and of which we only analysed 11 CpG positions, so we cannot conclude the exact influence of the overall methylation state of the promoter region).

## Conclusions

On the whole, the results presented here allow us to establish a profile of *KL* expression in PBCCs during atherosclerotic vascular disease, which is mediated by its promoter methylation. In addition, various associations with the inflammatory process are pointed out that reinforce the notion of a negative relationship between this anti-aging factor and a pro-inflammatory response, both at the systemic and vascular levels. These relationships allow to propose KL as a potential anti-inflammatory modulator that acts at different stages of the atherosclerotic damage, being necessary to investigate in depth the mechanisms underlying such associations in future studies.

## Supplementary Information

Supplementary Information.

## Data Availability

DNA sequences obtained by bisulfite sequencing are deposited in DDBJ (DNA Data Bank of Japan) with entry ID 621f54273a01a500641c43f1. Methylated C positions are represented as “m”.

## Abbreviations

AAA: Abdominal aortic aneurysm
CAD: Coronary artery disease
CRP: C-reactive protein
CVD: Cardiovascular disease
CVRF: Cardiovascular risk factors
DM: Diabetes mellitus
DNMT1: DNA Methyltransferase 1
DNMT3A: DNA Methyltransferase 3A
eGFR: Estimated glomerular filtration rate
HT: Hypertension
IL10: Interleukin 10
KL: α-Klotho
LDL: Low density lipoprotein
NFKB1: Nuclear Factor Kappa B Subunit 1
NLR: Neutrophil-to-lymphocyte ratio
PAD: Peripheral artery disease
PBCC: Peripheral blood circulating cell
sKL: Soluble α-Klotho
TIA: Transient ischemic attack
TNFα: Tumor necrosis factor α
