# Supplementary material for: Klotho expression in peripheral blood circulating cells is associated with vascular and systemic inflammation in atherosclerotic vascular disease
Source: Sci Rep. 2022 May 19;12:8422. doi: 10.1038/s41598-022-12548-z (PMC9120199; doi:10.1038/s41598-022-12548-z)
Supplement: Supplementary file 1 — Supplementary Information. [file 41598_2022_12548_MOESM1_ESM.docx]

| **A)**  **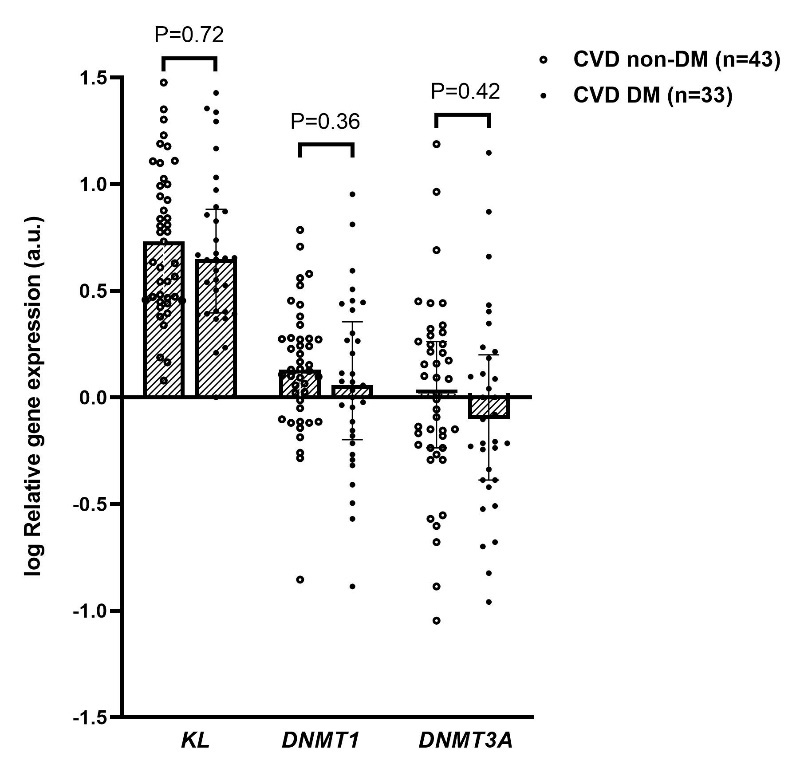** | **B)**  **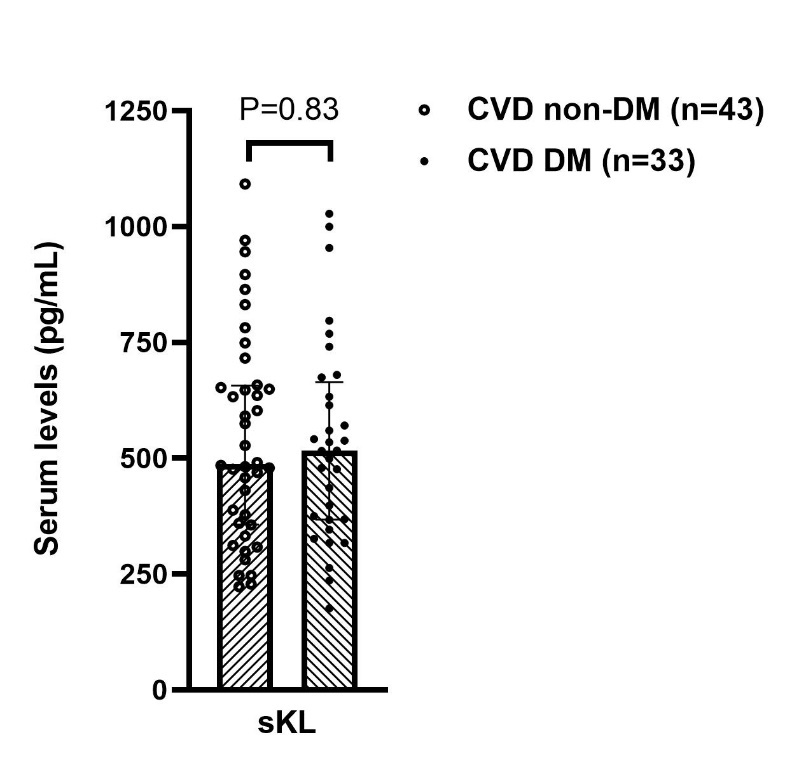** |
| --- | --- |
| **C)**  **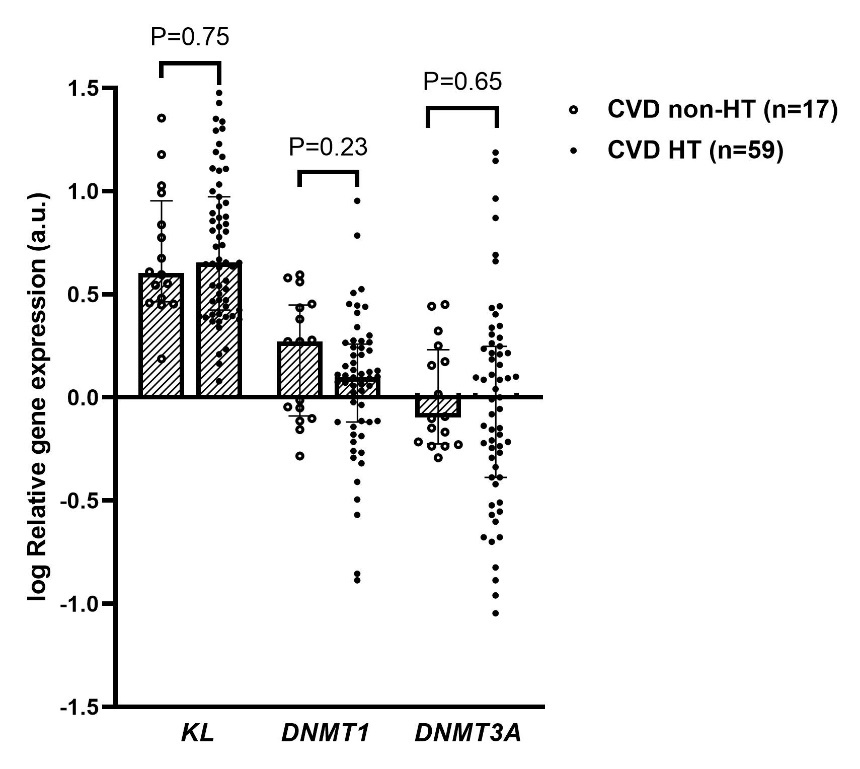** | **D)**  **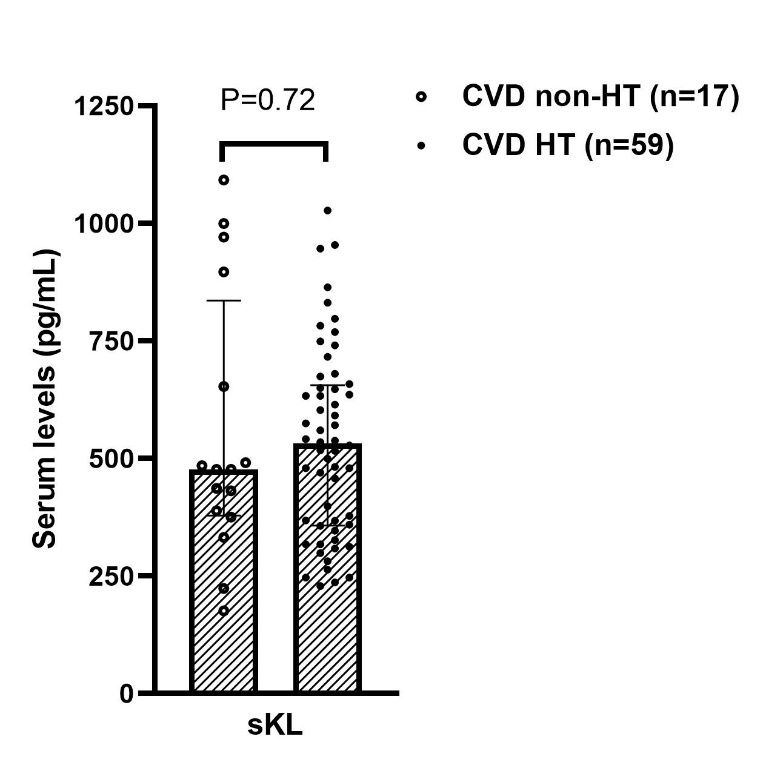** |
| **Supplementary Figure S1. KL levels according to the presence of diabetes mellitus (DM) (A, B) or arterial hypertension (HT) in CVD patients (C, D).** Relative gene expression levels of *KL, DNMT1* and *DNMT3A* in PBCCs (A, C). Serum levels of sKL (pg/mL) (B, D). a.u.: arbitrary units. Bars and range represent median and IQR. | |

| **** | **** | **** |
| --- | --- | --- |
| **** | **** | **** |
| **** | **** |  |
| **Supplementary Figure S2. Correlations between *KL* gene expression in PBCCs and covariates used in multiple regression analysis.** | | |

| **A)**  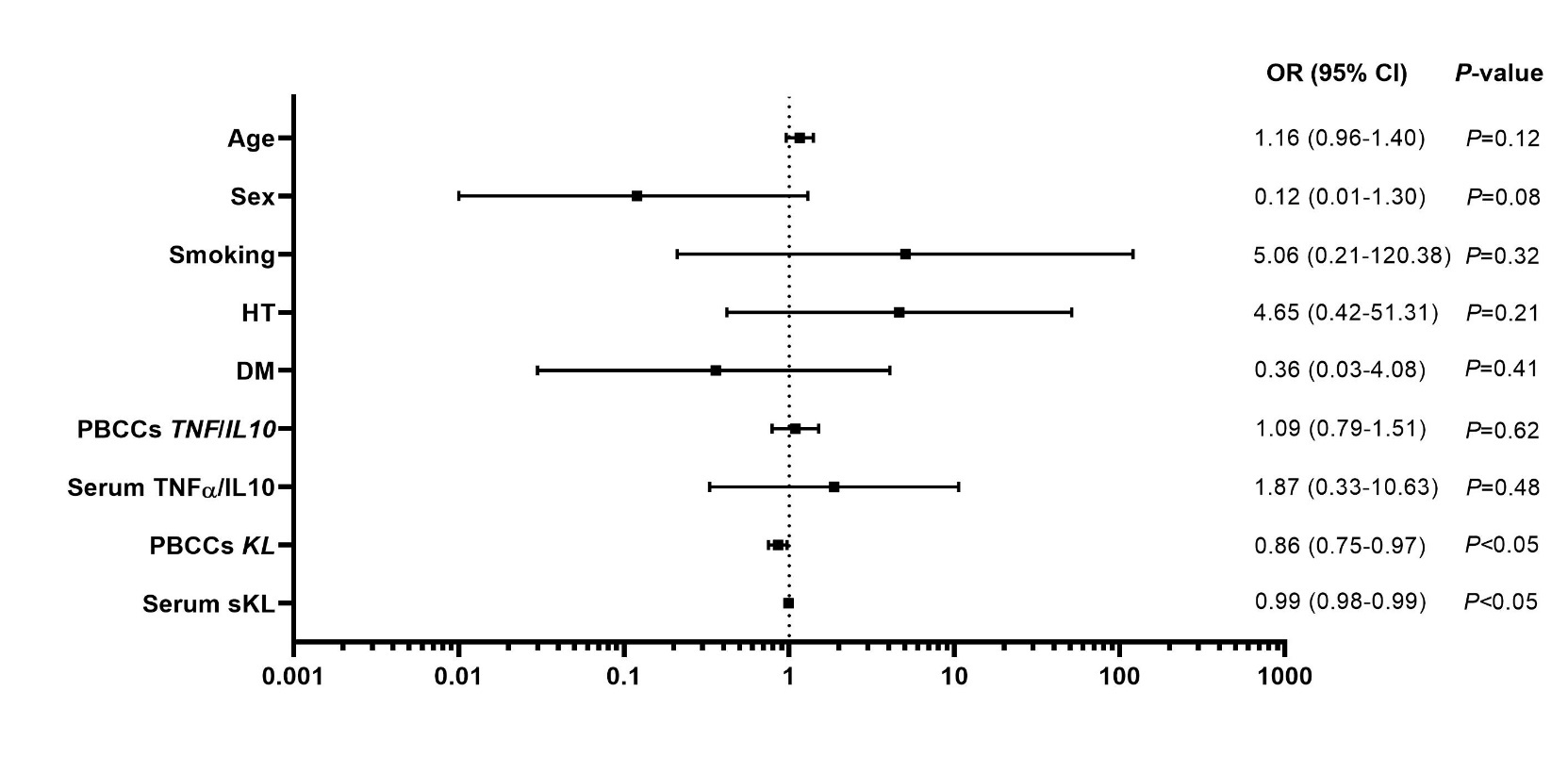 |
| --- |
| **B)**  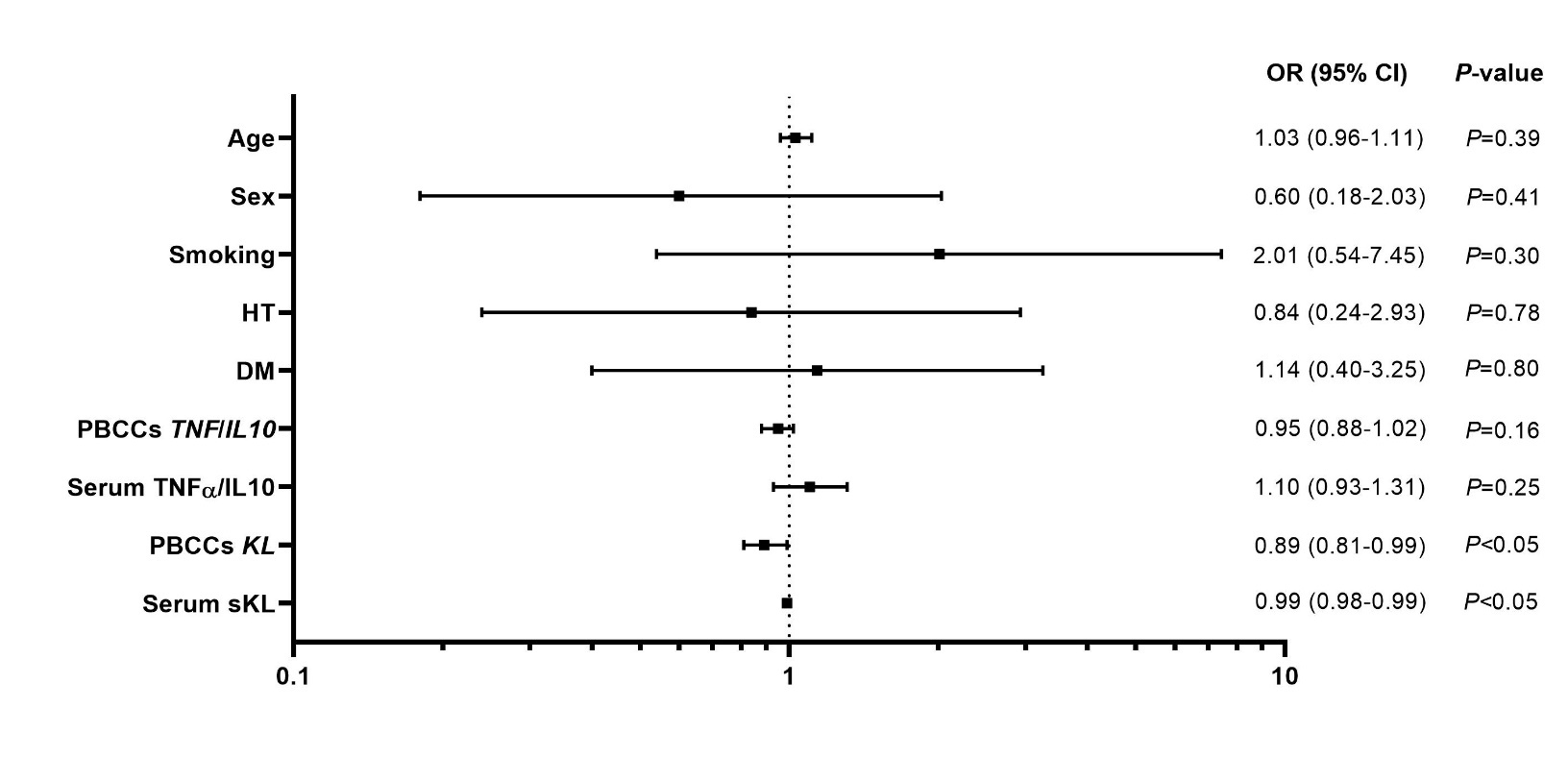 |
| **Supplementary Figure S3. Adjusted multivariate logistic regression models for the presence of CVD (A) or atherosclerotic plaque (B) displayed as the odds ratio (OR) with 95% confidence intervals (CIs).** |
